# A retrospective study of clinical signs and epidemiology of chronic valve disease in a group of 207 Dachshunds in Poland

**DOI:** 10.1186/1751-0147-55-52

**Published:** 2013-07-11

**Authors:** Magdalena Garncarz, Marta Parzeniecka-Jaworska, Michał Jank, Magdalena Łój

**Affiliations:** 1 Department of Veterinary Diagnostics and Pathology, Faculty of Veterinary Medicine, Warsaw University of Life Sciences, Nowoursynowska Str. 159c, Warsaw, Poland; 2 Department of Physiological Sciences, Faculty of Veterinary Medicine, Warsaw University of Life Sciences, Nowoursynowska Str. 159c, Warsaw, Poland

**Keywords:** Dog, Dachshund, Chronic valve disease, Cardiac murmur

## Abstract

**Background:**

Chronic mitral valve disease is frequently seen in the Dachshund. Dachshunds (n=207) made up 11.73% of the dogs admitted to the Cardiology Service at the Small Animal Clinic, Warsaw University of Life Sciences, Poland (first visits only).

**Results:**

Of these, 35 dogs had no clinically detectable heart disease while 172 had chronic valve disease with the mitral valve affected most often (130 dogs), both mitral and tricuspid valves infrequently (39 dogs) and rarely the tricuspid valve (3 dogs). Males were affected more frequently than females and the average age of dogs with chronic valve disease was 11.9 years for females and 11.3 years for males. A majority of the diseased Dachshunds were classified as ISACHC 2 (79), followed by ISACHC 1 (60). Most frequent clinical signs noted by owners included coughing, exercise intolerance, dyspnea and tachypnea. Heart murmurs were generally louder with increased disease severity; however there were 20 dogs in the ISACHC 1 group with no audible heart murmurs. The most frequent electrocardiographic abnormalities included an increased P wave and QRS complex duration, increased R wave amplitude and tachycardia. With increased disease severity, echocardiography revealed an increase in heart size. A higher ISACHC class was related to increased heart size (based on echocardiography) and increased percentage of patients exhibiting enlargement of both left atrium and left ventricle (based on radiography).

**Conclusions:**

The Dachshund is often affected by chronic mitral valvular disease with a late onset of associated clinical signs and few cardiac complications.

## Background

Due to their mild temperament, skills and great devotion to their owners, Dachshunds is a popular breed in Poland. Initially bred for hunting purposes, it is characterized by its’ brave nature and deep-rooted hunting instinct. The Dachshunds often fall ill due to chronic valvular disease (also known a valve disease or degenerative valve disease). Chronic valvular disease is the most frequent heart disease recognized in dogs throughout the world [1]. Research has shown that the disease affects over 90% of dogs over 13 years and 58% of dogs over 9 years of age [2]. The disease can occur in any breed, but is most commonly seen in small breed dogs with a predisposition in the Miniature Poodle, Cavalier King Charles Spaniel, Pomeranian, Yorkshire Terrier, Dachshund and Chihuahua [2]. Disease prevalence is in part related to breed popularity in different countries and even within different areas of one country. As the Dachshund breed is highly popular in Poland, the authors decided to retrospectively study clinical records for this breed with respect to heart disease as well as determine the prevalence within the breed to chronic mitral valve disease.

## Methods

A retrospective analysis was carried out on 40181 clinical charts of dogs admitted at the Small Animal Clinic, Faculty of Veterinary Medicine, Warsaw University of Life Sciences. The Cardiology Service saw 1875 dogs during that time, which was 4.66% of the Clinic’s total case load. Only first visit cases were included in the study. The analysis included information from the animal’s history, clinical examination, echocardiographic examination and where applicable electrocardiographic and radiographic examinations. The study group consisted of 207 Dachshunds. Dogs with recognized heart disease were classified according the International Small Animal Cardiac Health Council (ISACHC) classification scheme as class 1 – asymptomatic (1a – no cardiac enlargement, 1b with mild enlargement), class 2 - mild to moderate heart failure, and class 3 - severe heart failure (3a – no hospitalization required, 3b – hospitalization required). Heart murmurs in affected dogs were classified with respect to intensity according to a scale of 1–6 (Table 1).

**Table 1:** Heart murmur grading scheme

	
Grade 1	Very soft murmur heard only after very careful auscultation
Grade 2	Readily heard soft murmur
Grade 3	Moderately loud murmur not associated with palpable precordial thrill
Grade 4	Loud murmur, no or intermittent precordial thrill
Grade 5	Loud murmur associated with palpable precordial thrill; murmur not audible when stethoscope is lifted from thoracic wall
Grade 6	Loud murmur associated with palpable precordial thrill; heard when stethoscope is lifted from thoracic wall

All examinations were performed at rest without pharmacological restraint. A transthoracic echocardiographic (TTE) examination was performed in all dogs with an Aloka 4000 (after September 2005) or SC300 PANDION (before September 2005) ultrasound machines both equipped with a cardiology programs and 2.5 - 7-megahertz (mHz) sector transducers. The examinations were performed and interpreted by one of two doctors working in the Cardiology Service. Examinations were performed according to published norms [1,2], in some cases the examination was carried out on dogs in standing position [1,3]. Basic information from the echocardiographic examination allowing for a diagnosis of chronic valve disease included lesions of the mitral valve with regurgitation, lesion of the tricuspid valve with regurgitation, enlarged left atrium, increased left ventricular end-diastolic diameter, increased left ventricular end-systolic diameter, hyperdynamic left ventricular motion, hypodynamic left ventricular motion. Electrocardiographic examination (leads I,II, III, aVL, aVF and aVR) were performed with a BTL-08 MD machine. Radiological examination of the chest was performed with the G&E Prestige II X-ray machine.

### Statistical analyses

As a preliminary statistical analysis we involved frequency distribution and measurements of central tendency. Descriptive statistic results were presented as mean, standard deviation, median, minimum, and maximum or as number and percentage for numeric and categorical parameters, respectively. The relative risk modeling approach was used to perform verification of the association between explanatory variables and response. Cumulative logistic models were estimated to calculate the measures of relative risk between the categories due to the explanatory variables. These separate analyses can be interpreted as an estimate of a potential dose–response relationship between levels. The significance level was assumed as p < 0.05. Statistical analysis was performed using SAS (9.3).

Clinical data measured in each patient presented in Tables 2, 3 and 4 were analyzed statistically using one-way ANOVA test with Tuckey correction for multiple comparisons using GraphPad 6 software (GraphPad Prism, USA). The comparisons between two groups (males vs. females) were performed using unpaired t test with above mentioned software.

**Table 2:** The signalment and heart rate of Dachshunds submitted to cardiology services at the Small Animal Clinic, Faculty of Veterinary Medicine, Warsaw University of Life Sciences between November 2001 and October 2011 divided on the basis of heart disease severity (ISACHC class)

**Parameter**	**Class 1**	**Class 2**	**Class 3**
Number of dogs	60	79	33
Age (years) (mean ± SD)	11.0 ± 3.1^a^	12.1 ± 2.0^b^	11.4 ± 2.8^ab^
Body weight (kg) (mean ±SD)	9.4 ± 2.56	9.3 ± 1.87	9.7 ± 2.61
Heart rate (beats/min) (mean ±SD)	123 ± 19^a^	137 ± 18^b^	154 ± 32^c^
Sex (percentage of females in group) (%)	51.6	38.0	21.3

^abc^ Values in each row marked with different character are statistically different with p<0.05.

**Table 3:** Results of electrocardiography in Dachshunds submitted to cardiology services at the Small Animal Clinic, Faculty of Veterinary Medicine, Warsaw University of Life Sciences between November 2001 and October 2011 (number of positive dogs within the class)

	**No heart disease**	**ISACHC 1**	**ISACHC 2**	**ISACHC 3**
		**(n=12)**	**(n=20)**	**(n=11)**
	**(n=9)**			
Mean heart rate (bpm) (mean ± SD)	124 ± 26^a^	131 ± 25^a^	144 ± 27^a^	181 ± 37^b^
Tachycardia (>130/min)	3	6	16	11
Atrial premature depolarizations	0	0	1	0
Ventricular premature depolarizations	0	0	4	1
Atrial fibrillation	0	0	0	2
Increased P wave amp (>0,4 mV)	0	0	8	1
Increased P wave time (> 0,04 s)	2	2	15	4
Increased PR interval (>0,12 s)	1	0	2	0
Increased R wave amp (>2,5 mV)	1	1	9	2
Increased QRS complex time (>0,05 s)	3	6	5	11
Increased QT interval (>0,24s)	1	0	0	0

^ab^ Values marked with different character are statistically different with p<0.05.

**Table 4:** Selected results of echocardiography and radiography depending on severity of heart disease according to the ISACHC classification scheme in Dachshunds submitted to cardiology services at the Small Animal Clinic, Faculty of Veterinary Medicine, Warsaw University of Life Sciences between November 2001 and October 2011

**Parameter**	**Class 1**	**Class 2**	**Class 3**
LVDd, mm (mean ± SD)	2.98 ± 0.63^a^ (n=57)	3.78 ± 0.76^b^ (n= 75)	4.25 ± 0.53^c^ (n=32)
LVDs, mm (mean ± SD)	1.68 ± 0.47^a^ (n=57)	1.93 ± 0.44^b^ (n= 75)	2.12 ± 0.49^b^ (n=32)
LA/Ao	1.49 ± 0.24^a^ (n=58)	1.93 ± 0.44^b^ (n= 76)	2.51 ± 0.41^c^ (n=31)
LA, cm (mean ± SD)	2.38 ± 0.52^a^	3.15 ± 0.66^b^	3.85 ±0.70^c^
FS (%) (mean ± SD)	44.92 ± 8.49^a^	48.54 ± 7.08^b^	50.21 ± 7.55^b^
Left atrium enlargement on radiography (% of patients)	21.2 (n=33)	76.6 (n=47)	100.0 (n = 20)
Left ventricular enlargement on radiography (% of patients)	15.1 (n=33)	59.5 (n=47)	90.0 (n = 20)

*LVDd* left ventricular diameter diastole, *LVDs* left ventricular diameter systole, *LA/Ao* left atrial to aortic ratio, *LA* left atrium, *FS* fraction shortening. ^abc^ Values in each row marked with different character are statistically different with p<0.05.

## Results

The study population of Dachshund dogs made up 0.55% of all the dogs seen in the Small Animal Clinic during the allotted time and 11.73% of all first visits cases admitted to the Cardiology Service. The study group included 35 healthy Dachshund dogs (1.87% of all dogs seen at the Cardiology Service, 16.66% of all Dachshunds) as well as 172 Dachshund dogs with heart disease (9.33% of all dogs, 83.33% of all Dachshunds). Out of the 172 patients with recognized chronic heart disease 60 belonged to ISACHC class 1 (34.9%), 79 belonged to ISACHC class 2 (45.9%) and 33 belonged to ISACHC class 3 (19.2%). The signalment and heart rate (HR) of the patients in each ISACHC class are summarized in Table 2.

Dachshunds with heart disease (n=172) were diagnosed almost exclusively with chronic valve disease. There were two exceptions with one Dachshund diagnosed with subaortic stenosis and one with pulmonic valve dysplasia; however both dogs had concurrent chronic mitral valve disease. Dachshunds with chronic valve disease most often had lesions of the mitral valve (130 dogs), less often of both atrioventricular valves (39 dogs), and rarely of the tricuspid valve (3 dogs), which made up 75.58%, 22.67% and 1.71% of the affected dogs, respectively. Dogs with chronic valve disease were subdivided into ISACHC class 1 heart failure (mild), 2 (moderate) or 3 (severe). This classification is the equivalent of the recently published ACVIM classification scheme for canine chronic valvular heart disease respectively as stages B to D. According to this scheme healthy dogs susceptible to chronic mitral valve disease are classified as stage A (high risk for developing heart disease) [4,5].

Chronic valve disease occurred more frequently in males (n=104) than in females (n=68) showing a significant higher risk for the studied male population (p<0.001). The average age of females and males was 11.9 ± 2.7 years 11.3 ± 2.5 years, respectively. Also the average age of males and females in all ISACHC classes were similar – between 11 and 12 years of age, however the risk of the disease increased with age (7 years vs. 13 years of age, p<0.001).

Average body weight of diseased females was 8.9 ± 2.4 kg; whereas males had an average body weight of 9.8 ± 2.1 kg. (p=0.0107). However, there were no differences between the body weight of dogs (both males and females) belonging to different ISACHC classes (Table 2).

The clinical symptom most frequently noted by owners, regardless of ISACHC class, was coughing (Table 5). It was the only clinical symptom occurring in more than half of Class 2 patients (55.7%) and more than 75% of Class 3 patients and was the only symptom significantly related to chronic valve disease (p=0.0002). The increase in heart disease severity was accompanied by an increase in occurrence of exercise intolerance, dyspnea and tachypnea, which occurred in more than 50% of class 3 patients (Table 5). There were 14 dogs in ISACHC class 2 that were asymptomatic according to the owners, however after clinical examination and history evaluation, these dogs were classified as symptomatic. Dogs classified as ISACHC 1a (24 dogs) or 1b (7 dogs) had unspecific clinical symptoms such as cough, exercise intolerance or tachypnea, however additional tests (radiography and echocardiography) showed that these symptoms were in fact a result of chronic upper airway disease. Statistical analyses showed that clinical signs were noted by the owner more often with increasing severity of the heart disease when compared to asymptomatic or healthy dogs: tachypnoe (p<0.0001), exercise intolerance (p=0.0056), dyspnoe (p=0.0056), syncope (p=0.0311) and collapse (p=0.0098) (Table 5). However some owners did not recognize clinical signs of heart disease, specifically in the ISACHC 2 group.

**Table 5:** Clinical findings reported by owners depending on severity of heart disease according to the ISACHC classification scheme in Dachshunds submitted to cardiology services at the Small Animal Clinic, Faculty of Veterinary Medicine, Warsaw University of Life Sciences between November 2001 and October 2011

**Clinical findings**	**Class 1**	**Class 2**	**Class 3**
	**(n=60)**	**(n = 79)**	**(n=33)**
Cough (% of all patients)	30.0	55.7	75.7
Exercise intolerance (%)	23.3	41.7	63.3
Dysponoe (%)	20.0	41.7	60.8
Syncope (%)	1.7	8.8	15.1
Tachypnoe (%)	6.6	27.8	54.5
Collapse (%)	1.7	3.8	12.1

A typical heart murmur for chronic valve disease was noted in most dogs with a positive diagnosis, however there were 20 affected dogs with no audible murmurs present, all were dogs with mild disease belonging to ISACHC class 1 (Table 6). Heart murmurs occurred also in two dogs from the heart disease free group (group 0 – not classified as dogs with chronic valve disease / ACVIM Stage A).

**Table 6:** The occurrence and severity of heart murmur and heart failure in Dachshund submitted to cardiology services at the Small Animal Clinic, Faculty of Veterinary Medicine, Warsaw University of Life Sciences between November 2001 and October 2011 (% of positive dogs within the class)

**Heart murmur scale I-VI**	**No heart disease**	**Class 1**		**Class 2**	**Class3**	
Total n=207		1a	1b		3a	3b
	(n=35)	(n=44)	(n=16)	(n=79)	(n=31)	(n=2)
No murmur (%)	94.4	36.4	25.0	0	0	0
I (%)	2.8	4.5	6.2	1.4	0	0
II (%)	0	22.7	37.5	0	0	0
III (%)	2.8	36.4	31.3	54.5	16.2	50.0
IV (%)	0	0	0	40.5	29.0	0
V (%)	0	0	0	17.8	25.8	0
VI (%)	0	0	0	17.8	29.0	50.0

An electrocardiogram (ECG) was performed in 53 dogs (9 with no cardiac disease, 12 dogs ISACHC 1, 21 dogs in ISACHC 2 and 11 dogs ISACHC 3). Changes seen on the ECG are summarized in Table 3, included increased heart rate and more frequent signs of left heart enlargement with increase disease severity according to the ISACHC scheme. There were infrequent arrhythmias noted including one dog with supraventricular premature depolarizations (ISACHC 2) and 5 dogs with ventricular premature depolarization (4 dogs - ISACHC 2 and 1 dog - ISACHC 3). Two dogs (ISACHC 3) had atrial fibrillation.

The results of echocardiography and radiography clearly reflected the ISACHC classification (Table 4). Higher ISACHC class was accompanied by increased heart size (based on echocardiography) and increased percentage of patients exhibiting enlargement of both left atrium and left ventricle (based on radiography).

## Discussion

Chronic valve disease is characterized by myxomatous valvular degeneration, which most often leads to lesions of the mitral valve (75% of cases), infrequently the tricuspid valve (30% of cases) and sometimes both atrioventricular valves [1,3,6]. The associated valvular thickening and abnormal leaflet motion leads to incomplete coaptation of the valvular leaflets and to regurgitation. This in turn causes left atrial (or correspondingly right atrial) and left (right) ventricular enlargement. In the course of the disease it is common to also note chordae tendinae lengthening and sometimes rupture [3,7]. Histological changes include deposition of mucopolysaccharides in the spongiosa layer of the valve leaflets [1,2]. The pathogenesis of the disease is unknown, however there has been attempts to link it to systemic connective tissue diseases because both entities have a high prevalence in the same, small breed dogs [1]. Others have suggested that endothelin plays a role in the pathologic process [1,2]. Regardless of cause, studies have shown that biomarkers such as cardiac troponin I [8] and NT-proBNP [9] increase with increased disease severity. There is a high breed predisposition to chronic valvular disease and therefore a genetic predisposition is highly suspect [1-3] with a clear majority of affected breeds being of small size [10]. One study on the epidemiology and mode of inheritance in four families of Dachshunds excluded an autosomal recessive, X-linked dominant and X-linked recessive mode of inheritance [11,12]. The Cavalier King Charles Spaniel has a great predisposition to the disease and has therefore been extensively studied showing a clear heritability, although specific genetic changes are yet to be identified [6,13]. This breed is slowly becoming popular in Poland, however their numbers are still too low for a good study population.

Our study showed a high incidence of mitral valve disease while the incidence of tricuspid disease was lower and concurrent mitral and tricuspid valve disease higher than previously reported [1,14]. This might be due to our study population of solely Dachshunds as published data [1,14] are for several breeds or the regional disease characteristics. However it should be noted that published studies on distribution of myxomatous lesions with respect to the mitral and tricuspid valves date back to the 1960-70′s and are often based on histopathology. This study did not encompass histopathological examination of affected valves, and although echocardiography showed clear leaflet lesions and best efforts were made to non-invasively rule out primary respiratory disease leading to pulmonary hypertension, these potential reasons for significant tricuspid regurgitation cannot be fully excluded.

Our study also showed a higher prevalence of chronic valve disease in males than in females, which is in line with the general tendency observed in other studies [1,11]. The ratio of female to males in the Clinic’s general population was almost 1:1 during the studied period so it appears that males are generally more prone to valvular disease, In our study there were more affected males in ISACHC class 2 and class 3 patients, which could suggest the more severe course of the disease in males. The difference between the age of disease onset in males and females was insignificant but both sexes showed an age-related increased risk of disease. This risk is extremely high in dogs older than 13 years, when compared to adult dogs until 7 years of age.

Our study showed that the occurrence of symptoms such as cough, dyspnoe, tachypnoe, exercise intolerance and syncope are related with quite advanced heart disease. However only cough was highly suggestive for chronic valve disease.

Chronic valve disease is often recognized during routine clinical examination on the basis of a typical heart murmur heard particular over the mitral valve area. A direct relationship has been shown between the intensity of heart murmurs and the severity of heart disease, specifically chronic valve disease [1,2,13,15] and it has been shown that dogs with heart murmurs at are higher risk for a cardiac associated death [16]. In our study the presence and intensity of heart murmurs generally reflected the ISACHC classification and therefore was a good indicator of disease severity (Table 6). However lack of heart murmur did not rule out chronic valvular disease. A recent study also showed a high number affected animals with mitral valve disease without heart murmurs during clinical examination and this warrants further studies to assess disease development [17]. There was one dog with a 3/6 heart murmur that had no heart disease; a finding probably due to functional heart murmurs, including changes in blood morphology, increased body temperature, turbulent blood flow related to stress tachycardia. This particular dog had an elevated body temperature, an increase in heart rate (160 bpm) and was subsequently diagnosed with *Babesia canis* infection. The second dog with a 1/6 heart murmur and no heart disease was subsequently diagnosed with Cushing’s disease, but no clear reason for this murmur was diagnosed.

There was a discrepancy between the information acquired from the owners and the clinical examination in 14 dogs (8%) as owners did not notice clinical symptoms that were evident at clinical examination. This could be a result of treatment leading to an improved clinical state.

One of the most characteristic features of heart disease in dogs is increased heart rate. In our studies heart rate were measured both traditionally by auscultation (Table 2) and as part of the electrocardiography (Table 3). Both types of measurements clearly show increased heart rate corresponding to increased ISACHC class number; however there is a difference between the values obtained traditionally and during electrocardiography. All the values obtained using electrocardiography were higher and in case of ISACHC Class 3 patients the difference was about 20% higher than the value obtained during standard clinical examination.

## Conclusion

Chronic mitral valve disease is prevalent in the Polish Dachshund breed and occurs with a late onset of associated clinical signs and few cardiac complications. The study showed chronic mitral valve disease occurred more often in males than females, A lack of heart murmur in the Dachshund breed does not rule out chronic valve disease, specifically in dogs classified as ISACHC 1 (ACVIM B). Increased severity and frequency of coughing is a good indicator of mitral valve disease progression in Dachshunds, however primary respiratory disease must be first ruled out or addressed, especially in the milder forms of disease.

## Competing interests

The authors declare that they have no competing interests.

## Authors’ contributions

MG conceived and participated in the design of the study, carried out patient examinations and drafted the article. MJ cconceived and participated in the design of the study, took part in drafting the initial article and performed the statistical analysis. MŁ took part in drafting the initial article and collected pertinent literature. MPJ did the patient examinations and took part in drafting the article. All authors read and approved the final manuscript.
